# The Prevalence of Trachomatous Trichiasis in People Aged 15 Years and Over in Six Evaluation Units of Gaoual, Labé, Dalaba and Beyla Districts, Guinea

**DOI:** 10.1080/09286586.2023.2192269

**Published:** 2023-06-28

**Authors:** Midiaou M. Bah, Fatoumata Sakho, André Goepogui, Luc C. Nieba, Abdourahim Cisse, Paul Courtright, Anna J. Harte, Clara Burgert-Brucker, Cristina Jimenez, Pierre L. Lama, Michel Sagno, Ana Bakhtiari, Sarah Boyd, Anthony W. Solomon, Michaela Kelly, Fiona James, Moise S.D. Tenkiano, Emma M. Harding-Esch, Boubacar M. Dicko

**Affiliations:** a Neglected Tropical Diseases, Sightsavers, Conakry, Guinea; b Ministère de la Santé, National Programme for the Control of Neglected Tropical Diseases, Conakry, Guinea; c Kilimanjaro Centre for Community Ophthalmology, Division of Ophthalmology, University of Cape Town, Cape Town, South Africa; d Clinical Research Department, London School of Hygiene & Tropical Medicine, London, UK; e Neglected Tropical Diseases, RTI International, Washington, District of Columbia, USA; f Neglected Tropical Diseases, Sightsavers, Haywards Heath, UK; g International Trachoma Initiative, Task Force for Global Health, Atlanta, Georgia, USA; h Department of Control of Neglected Tropical Diseases, World Health Organization, Geneva, Switzerland

**Keywords:** Elimination, epidemiology, Guinea, trachomatous trichiasis, TT-only surveys

## Abstract

**Purpose:**

Trachoma is a public health problem in 42 countries. Inflammation associated with repeated ocular infection with *Chlamydia trachomatis* can cause the eyelid to scar and turn inwards, resulting in the eyelashes rubbing against the eyeball, known as trachomatous trichiasis (TT). In Guinea, baseline surveys conducted in 2013 reported inflammatory trachoma prevalences below the World Health Organization (WHO) threshold for elimination, but TT prevalences above threshold. Given this epidemiological context and time since baseline survey, TT-only surveys were conducted in selected districts to determine current TT prevalence. The results of this study provide critical data for assessing Guinea’s achievement of trachoma elimination targets.

**Methods:**

Four health districts, consisting of six evaluation units (EU), were surveyed. In each EU, field teams visited 29 clusters with a minimum 30 households included in each. Participants aged≥15 years were examined by certified graders trained to identify TT and determine whether management had been offered.

**Results:**

A total of 22,476 people were examined, with 48 TT cases across the six EUs identified. Five of six EUs had an age-and-gender adjusted TT-prevalence unknown to the health system less than 0.2%, whereas one EU, Beyla 2, had an adjusted TT prevalence of 0.24%.

**Conclusion:**

These TT-only surveys, along with findings from other trachoma interventions, suggest that Guinea is close to achieving elimination of trachoma as a public health problem. This study demonstrates the value of undertaking TT-only surveys in settings where baseline surveys indicated active trachoma prevalences below WHO elimination threshold, but TT prevalences above it.

## Introduction

Trachoma is a bacterial eye disease caused by *Chlamydia trachomatis* and is a public health problem in 42 countries, primarily in lower- and middle-income countries; many in sub-Saharan Africa. ^1, 2^ Infection is associated with active (inflammatory) trachoma, most commonly in pre-school children. Factors associated with transmission are unclean faces and limited availability or use of latrines, which can increase the number of *C. trachomatis* -carrying flies, *Musca sorbens*.^3–10^

Trachoma is the leading infectious cause of blindness worldwide.^11^ Repeated infection and associated episodes of active trachoma can lead to scarring of the tarsal conjunctiva,^12,13^ which can result in the upper eyelid turning inwards, causing the eyelashes to rub against the eyeball. This painful state is called trachomatous trichiasis (TT) and can damage the cornea, leading to opacities that can result in blindness.^14^ The prevalence of TT increases with age; it is rarely present before the age of 15 years.^15,16^ As of June 2022, over 125 million people worldwide were estimated to be at risk of blindness due to trachoma.^1^ The SAFE strategy, developed by the World Health Organization (WHO) for the elimination of trachoma as a public health problem, includes: TT Surgery (S), Antibiotics (A), Facial Cleanliness (F) and Environmental improvement (E).^17,18^ In trachoma-endemic areas, elimination as a public health problem requires evidence of a trachomatous inflammation—follicular (TF) prevalence below 5% in children aged 1–9 years and a prevalence of TT unknown to the health system below 0.2% in adults aged 15 years and above.

In Guinea, national baseline mapping of trachoma took place in 31 health districts from 2011–2016; 18 health districts required implementation of SAFE.^19^ There is limited information on the implementation of F and E activities in Guinea; a study on water, sanitation and hygiene conducted by the Guinea health ministry in collaboration with Sightsavers in 2020 demonstrated the existence of small-scale interventions delivered to rural communities throughout the country, however this activity was not related to the trachoma elimination programme nor was it implemented district-wide.^20^ Since July 2021, Sightsavers has been supporting a water and sanitation behaviour change programme contributing to the reduction of TF, through the Guinean NGO Tinkisso. Surgical outreach campaigns have been implemented across the country since 2013. Individuals identified with TT during the baseline surveys were offered surgery, and in areas with high TT prevalence, house-to-house screening was conducted using local volunteers trained to identify TT cases, which were later confirmed by ophthalmic nurses. Based on the 2011–2016 baseline surveys, the country-wide TT backlog was estimated at 32,737; the most up-to-date estimate (January 2023) is a backlog of 7,774 cases.

Since 2013, antibiotic interventions have been implemented following WHO recommendations.^21^ Sixteen districts have benefited from mass drug administration (MDA) of azithromycin (tablets and syrup) and tetracycline eye ointment. Two districts, Koundara and Mali, did not receive MDA, as the original surveys were considered outdated and potentially unreliable. Instead, they underwent re-survey in 2017 and 2018 using a standard trachoma prevelance survey methodology,^22^ both returning TF and TT prevalences below elimination threshold.^23^ Impact surveys were carried out in the 16 MDA-treated health districts between 2017–2019, followed by surveillance surveys in 2020. The results of the latest surveys demonstrated a TF prevalence in 1–9-year-olds under 5% in all districts, however TT prevalence was above the WHO elimination threshold in eight districts.

Given the long duration since baseline surveys, it is likely that the original TT prevalence estimates do not reflect the current status. Furthermore, since baseline TF prevalence was below elimination threshold, further surveys collecting data on TF prevalence in 1–9-year-olds are unnecessary. For these reasons, TT-only surveys were recommended in four health districts (Gaoual, Labé, Dalaba and Beyla) in which TF prevalence was<5% and TT prevalence was≥0.2% in baseline surveys in 2013–2014 to determine if additional TT interventions are recommended.^19^ TT-only surveys are a tool that countries can use to support validation of elimination of TT as a public health problem.^24^ TT-only surveys focus specifically on adults aged≥15 years and have larger sample sizes than normal trachoma prevalence surveys, which recruit fewer adults and therefore estimate TT prevalence with less precision. This paper is one of the first reporting on TT-specific re-surveying in order to determine TT intervention needs and provide data for trachoma elimination dossier preparation for submission to WHO.^25^

## Materials and methods

### Ethical considerations

Ethical approval for this study was provided by the Guinea Ministère de la Santé (033/CNERS/21) and the London School of Hygiene & Tropical Medicine, UK (16105). Every participant was provided with a clear explanation of the purpose and use of their data. Informed consent was required from individuals surveyed and was recorded in Android smartphones. Barrier measures for the prevention of SARS-CoV-2 transmission were implemented from 2020 onwards as per the standard operating procedures developed by Guinea according to WHO guidance,^26^ including the use of face masks and disposable gloves, which were changed between each individual examination.

### Study design and participant selection

This was a cross-sectional study to assess the prevalence of TT in the populations of four health districts: Gaoual, Dalaba, Labé and Beyla. Cluster random sampling was undertaken at two levels – villages and households – with a single visit made to each selected village. The WHO-recommended standardized methodology outlines the requirements for trachoma surveys, in which the evaluation unit (EU) is defined as a local health administrative unit with a population of 100,000–250,000 people.^27^ Therefore, two health districts with populations larger than 250,000, Labé and Beyla, were each split into two separate EUs (Labé 1, Labé 2; Beyla 1, Beyla 2; Tables 1 and 2).

**Table 1.** Survey demographics of the six evaluation units (EU) surveyed using the TT (trachomatous trichiasis)-only methodology, Guinea, Jan-Feb 2021.

EU code	Region	District	EU name	2020 population	Number of clusters surveyed	Number of households surveyed	Number of≥15-year-olds enumerated	Number of≥15-year-olds examined (%)	Number of females examined (%)
50169	Boke	Gaoual	Gaoual	231,483	29	882	3887	3885 (99.9)	2159 (55.6)
50170	Mamou	Dalaba	Dalaba	159,754	29	882	3724	3721 (99.9)	2143 (57.6)
50171	Labe	Labe	Labe 1	224,982	29	888	3743	3734 (99.8)	2214 (59.3)
50172	Labe	Labe	Labe 2	154,995	29	879	3704	3698 (99.8)	2224 (60.1)
50173	N’Zerekore	Beyla	Beyla 1	187,565	29	884	3620	3609 (99.7)	2128 (59.0)
50174	N’Zerekore	Beyla	Beyla 2	204,369	29	884	3836	3829 (99.8)	2248 (58.7)

**Table 2.** Baseline (2013–2014) and TT-only (2020–2021) survey results, Guinea.

Region	District	Evaluation Unit	Baseline surveys 2013–2014^a^		TT-only surveys 2021		
			TF prevalence (95% CI)	TT prevalence (95% CI)	Number of≥15-year-olds with TT (n, % female)	Age- and gender-adjusted TT prevalence (95% CI)	Age- and gender-adjusted TT unknown to the health system prevalence (95% CI)
Boke	Gaoual	Gaoual	3.6 (2.8–4.5)	0.6 (0.3–1.0)	10 (7, 70.0)	0.13 (0.05–0.23)	0.10 (0.03–0.21)
Mamou	Dalaba	Dalaba	2.0 (1.2–3.1)	0.2 (0.1–0.6)	0	0	0
Labe	Labe	Labe 1	2.7 (1.8–3.9)	0.8 (0.4–1.4)	3 (1, 33.3)	0.05 (0.0–0.12)	0.03 (0.00–0.08)
Labe	Labe	Labe 2	2.7 (1.8–3.9)	0.8 (0.4–1.4)	11 (8, 72.7)	0.15 (0.03–0.32)	0.13 (0.02–0.30)
N’Zerekore	Beyla	Beyla 1	2.4 (1.8–3.1)	0.3 (0.1–0.6)	10 (7, 70.0)	0.14 (0.04–0.23)	0.14 (0.04–0.23)
N’Zerekore	Beyla	Beyla 2	2.4 (1.8–3.1)	0.3 (0.1–0.6)	14 (9, 64.3)	0.24 (0.09–0.41)	0.24 (0.09–0.41)

Note: ^a^ Data from Global Trachoma Mapping Project (GTMP)^19^. CI: Confidence interval. TF: trachomatous inflammation—follicular. TT: trachomatous trichiasis. TT is defined as at least one eyelash from the upper eyelid touching the eyeball, or evidence of recent epilation of in-turned eyelashes from the upper eyelid.^32^ A case of TT unknown to the health system indicates that the participant has not received an offer of epilation or surgery from a healthcare worker, or responded “I don’t know” to the questions asked regarding TT management.^27^ Trichiasis is the presence of at least one eyelash from either eyelid touching the eyeball, or evidence of recent epilation of in-turned eyelashes from either eyelid.

All participants were aged≥15 years, had lived for at least a month in the selected village, and belonged to a selected household. Participant selection methods and number of participants required for TT-only surveys have been standardized, as described elsewhere.^28^ In brief, the sample size was determined by the single population proportion for precision formula that includes the expected prevalence (0.2%), standard deviation corresponding to 95% confidence intervals (1.96), the design effect size (1.47) and the margin of error (0.2%), resulting in a sample size of 2,818 people to examine. This value is then multiplied by the estimated non-response inflation factor (1.2), resulting in a final sample size of 3,382 individuals to enumerate per EU. In Guinea, the number of residents aged≥15 years per household is estimated to be 4,^29^ resulting in 846 households required in total. A survey team can visit~30 households per cluster per day, meaning a total of 29 clusters per EU were required to meet the sample size.

### Training and clinical examinations

Trachoma graders and recorders were trained and certified following the methods developed by Tropical Data (www.tropicaldata.org).^30^ In brief, graders were given two days of training on examination techniques including proper handwashing, everting the eyelids, looking for surgical scars or epilation, and identifying the clinical signs of trichiasis (upper and lower eyelid, separately). Graders were tested using Objective Structured Clinical Evaluation techniques to quality-assure clinical evaluation. Recorders were trained to enter data into Android smartphones using the Tropical Data software. All consenting adults were seen by a certified grader. The clinical examination was performed with binocular magnifying loupes (2.5×) and under adequate lighting (preferably daylight, with a torch used in low light conditions). Each eyelid was examined separately. All clinicals signs were graded according to the WHO simplified trachoma grading system.^31,32^

The graders first looked for the presence of eyelashes touching the eyeball or signs of eyelash removal from the upper eyelids (TT) and on the lower eyelids (lower eyelid trichiasis).^32^ Data on lower eyelid trichiasis were collected to enable comparison with data collected before the TT definition was modified at the 4^th^ Global Scientific Meeting on Trachoma in 2018,^24^ and to support countries with their trichiasis management plans. However, data on lower eyelid trichiasis are not used to determine if TT elimination has been achieved. For individuals with trichiasis in either eyelid, the examiner everted the upper eyelid, and sought evidence of a surgical scar. Individuals with trichiasis (upper or lower eyelid) were then asked questions about health management for the trichiasis; an individual was defined as “known to the health system” if the participant answered yes to the question of having had or been offered surgery or epilation, or if the participant answered no to the question of having had surgery, but the grader saw a surgical scar and subsequent discussion confirmed that there was a history of eyelid surgery.^33^

Participants with TT were offered free TT surgery, the scheduling of which was done in agreement between the participant and the village and health authorities. Participants with TF were treated with 1% tetracycline ophthalmic ointment (to be applied twice daily for 6 weeks) or a single oral azithromycin dose (1 g for adults).

### Definitions

TT is defined as at least one eyelash from the upper eyelid touching the eyeball, or evidence of recent epilation of in-turned eyelashes from the upper eyelid.^32^ TT “unknown to the health system” indicates TT for which the participant had not received an offer of epilation or surgery from a healthcare worker, or in a participant who responded “I don’t know” to the questions asked regarding TT management and did not have any evidence of a surgical scar.^27^

### Data recording, storage and analysis

Data were recorded in the Tropical Data app using Android smartphones. Data were uploaded to a secure central server, enabling the dedicated Tropical Data data team to clean and validate the data in partnership with the Programme National de Lutte contre les Maladies Tropicales Négligées (PNLMTN). Standardized data analyses using automated algorithms were conducted to calculate the age- and gender-adjusted prevalence of TT.^22^

Further data association analyses were performed to determine the relationship between age, gender and the prevalence of all TT known or unknown to the health system using the glmer R package (R Core Team, 2021). Models including household, cluster and EU as random effects were tested for best fit using maximum likelihood tests. Odds ratio (OR) values for the likelihood of TT prevalence were determined for each age bracket and gender. Individual age values were grouped into age brackets of 15–45, 46–75, and 75+ years; these age brackets were chosen as the models did not run with smaller age bands^34^ due to the low numbers of TT cases.

## Results

A total of 22,514 people were enumerated, with 22,476 (99.8%) examined from January to February 2021. A mean of 58% of participants per EU were women. In each EU, 29 clusters and~883 households were examined (Table 1).

**Figure 1.**
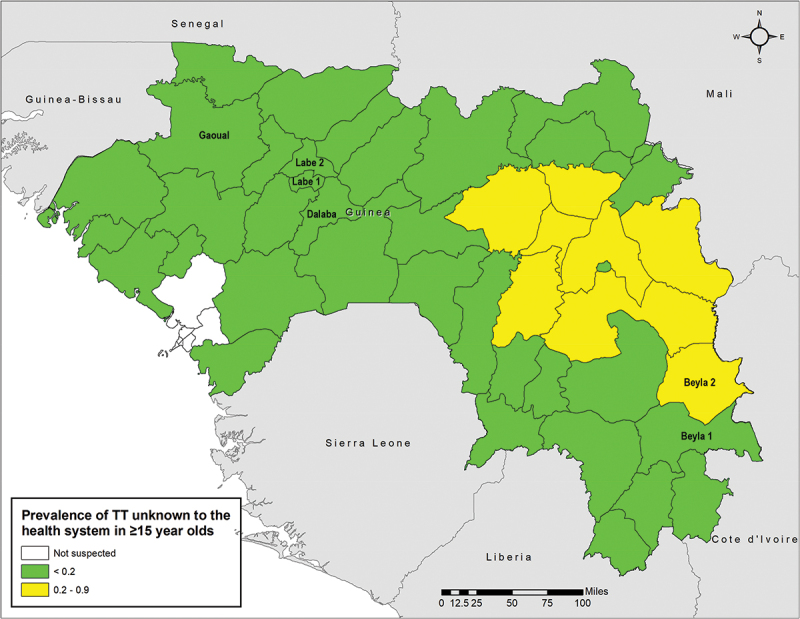
Age- and gender-adjusted prevalence of trachomatous trichiasis (TT) unknown to the health system in Guinea, as of 2021. Evaluation units (EU) measured in this survey are identified by the EU name (6 EUs) (2020–2021). The boundaries and names shown and the designations used on this map do not imply the expression of any opinion whatsoever on the part of the authors, or the institutions with which they are affiliated, concerning the legal status of any country, territory, city or area or of its authorities, or concerning the delimitation of its frontiers or boundaries.

There were 48 cases of TT across all six EUs, with one EU, Dalaba, in Mamou Region, having zero cases (Table 2). A larger proportion of women than men had TT, and only five of the 48 cases (10.4%) were known to the health system, three in Labe 2, and one each in Labe 1 and Gaoual. There were two cases where trichiasis was only present on the lower eyelid. The age-and-gender adjusted TT-prevalence unknown to the health system was<0.2% in five of six EUs (Figure 1); the remaining EU, Beyla 2, had an adjusted TT prevalence unknown to the health system of 0.2% (95% confidence interval (CI) 0.1–0.4).

### Association analysis

Models testing the three random effects of household, cluster and EU were unable to converge with the inclusion of household and EU; therefore, only cluster was used. P-values indicated that both age and gender were strongly associated with TT prevalence (age: *p* < .001; gender: *p* < .05). The OR for gender indicated over double the odds of TT in women compared to men (OR: 2.1, 95% CI 1.1–3.8), and individuals aged 46–75 or >75 years had greater odds of having TT relative to those aged 15–45 years (46–75, OR: 22, 95% CI: 7–74; >75, OR: 95, 95% CI: 27–330).

## Discussion

Guinea has been working towards trachoma elimination since the start of nationwide trachoma surveys in 2011. Excellent progress has been made with regards to decreasing the prevalence of TF to below the 5% elimination threshold around the country; all previously endemic health districts have completed their required number of antibiotic MDA rounds and the 2021 TF prevalence estimates in all EUs were<5%.^23^ Guinea is currently in the process of completing the final pre-validation surveillance surveys in the EUs that underwent MDA treatment and predicts that all standard surveys will be completed by 2023, security permitting.

In the six EUs included in this TT-only survey series, five had an adjusted prevalence of TT unknown to the health system less than the WHO elimination threshold of 0.2%. In these EUs, the management of TT should be undertaken within routine eye health services.^35^ One EU, Beyla 2, had a prevalence of TT unknown to the health system of 0.24%, indicating that public health-level TT surgery services are needed.

The 2013–2014 baseline estimates of TT prevalence in the surveyed EUs were higher than the TT-only survey results in this study (Table 2). Since approximately eight years have elapsed since the baseline surveys, it is possible that this reduction is due to surgical treatment campaigns: the Guinea Ministère de la Santé Neglected Tropical Diseases programme reports that the national number of cases operated in 2015, 2016, 2017, 2018 and 2019 were, respectively: 3689, 936, 1119, 676 and 468; however the districts included in this study were not targeted for public health interventions. Individuals identified with TT in the original baseline surveys were offered surgery at the time. Of the five individuals who had TT and were known to the health system in our study, four had been offered and undergone surgery, and the remaining person had accepted surgery but had not yet received it, indicating TT surgery is or has been available in at least three of the EUs studied here. Since the baseline prevalence of TF in these areas was very low,^19^ the current low TT prevalence may also be the result of few incident cases, combined with a proportion of individuals with TT having died.

The one EU with a TT prevalence above the elimination threshold, Beyla 2, in the northern section of the health district of Beyla, is located in southeast Guinea, bordering Côte d’Ivoire. While the Côte d’Ivoire districts of Odienne and Koro that are adjacent to Beyla 2 are both endemic for trachoma (2015 baseline TF > 10%), neither of these districts has had surveys reporting TT as a public health problem.^23^ Two out of three districts that are adjacent to Beyla 2 in Guinea demonstrate a prevalence of TT below the WHO threshold for elimination, with the exception of Kankan 3 (0.4%, as of December 2021) (Figure 1).

The association analysis results concur with the outcomes of many other studies,^16,36,37^ in that women were twice as likely to have TT compared to men, and people aged>46 years were significantly more likely to have TT relative to those aged 15–45 years. The higher prevalence of TT in women is thought to be largely due to them spending more time in close proximity with young children, the main reservoir of ocular *C. trachomatis* infection.^36,37^ Increasing age is an expected association of TT due to the pathogenesis of trachoma: it is the repeated infections over time which lead to the development of conjunctival scarring, and in turn to TT. Therefore, older people are more likely to have experienced a sufficient number of *C. trachomatis* infections to develop TT, relative to younger people.^12,13^

In five of the surveyed EUs, TT management should be integrated within the routine clinical services. To ensure that Guinea reaches elimination, it is recommended that Beyla 2 undergoes TT-specific public health interventions. The current estimated TT backlog in Beyla district is 391. Thirty-one trichiasis cases were managed in 2015 (prior to the TT-only survey), however there were no cases managed after this point. If a public health approach to TT is implemented, Beyla 2 will require documentation of full geographic coverage of case finding and outreach services, or a subsequent TT-only survey to demonstrate that elimination has been achieved.

The cost of running TT-only surveys is estimated at $9,707 per EU,^38^ which can be a barrier to documenting trachoma elimination. Many of the standard surveys are supported by external partners, and countries may struggle to undertake TT-only surveys without additional funding. The benefit of these surveys is clear however, when there is a likelihood that old baseline data is not an accurate reflection of current TT prevalence. Geospatial analysis may be a useful approach for providing more precise TT prevalence estimates either through more efficient survey design or generating estimates using existing survey data, if appropriate.^39^

Guinea is currently working on a transition plan to ensure the availability of incident TT case management in formerly endemic districts in-line with WHO elimination criteria. At the present time, there are seven EUs in Guinea that have a prevalence of TT unknown to the health system≥0.2% requiring interventions in order to achieve elimination of TT as a public health problem. Completion of these interventions and demonstration of below elimination threshold prevalence should provide the final data needed for dossier submission to WHO for trachoma elimination validation.
